# A Common Variant in *CLDN14* is Associated with Primary Biliary Cirrhosis and Bone Mineral Density

**DOI:** 10.1038/srep19877

**Published:** 2016-02-04

**Authors:** Ruqi Tang, Yiran Wei, Zhiqiang Li, Haoyan Chen, Qi Miao, Zhaolian Bian, Haiyan Zhang, Qixia Wang, Zhaoyue Wang, Min Lian, Fan Yang, Xiang Jiang, Yue Yang, Enling Li, Michael F. Seldin, M. Eric Gershwin, Wilson Liao, Yongyong Shi, Xiong Ma

**Affiliations:** 1 State Key Laboratory for Oncogenes and Related Genes, Division of Gastroenterology and Hepatology, Renji Hospital, School of Medicine, Shanghai Jiao Tong University, Shanghai Cancer Institute, Shanghai Institute of Digestive Disease, 145 Middle Shandong Road, Shanghai 200001, China; 2 Bio-X Institutes, Key Laboratory for the Genetics of Developmental and Neuropsychiatric Disorders (Ministry of Education), Shanghai Jiao Tong University, Shanghai 200030, China; 3 Department of Biochemistry and Molecular Medicine, University of California at Davis, Davis, CA 95616, USA; 4 Division of Rheumatology, Allergy and Clinical Immunology, University of California at Davis School of Medicine, Genome and Biomedical Sciences Facility, 451 Health Sciences Drive, Suite 6510, Davis, CA 95616, USA; 5 Department of Dermatology, University of California San Francisco School of Medicine, 1701 Dividadero Street, San Francisco, CA 94415, USA

## Abstract

Primary biliary cirrhosis (PBC), a chronic autoimmune liver disease, has been associated with increased incidence of osteoporosis. Intriguingly, two PBC susceptibility loci identified through genome-wide association studies are also involved in bone mineral density (BMD). These observations led us to investigate the genetic variants shared between PBC and BMD. We evaluated 72 genome-wide significant BMD SNPs for association with PBC using two European GWAS data sets (n = 8392), with replication of significant findings in a Chinese cohort (685 cases, 1152 controls). Our analysis identified a novel variant in the intron of the *CLDN14* gene (rs170183, *P*_fdr_ = 0.015) after multiple testing correction. The three associated variants were followed-up in the Chinese cohort; one SNP rs170183 demonstrated consistent evidence of association in diverse ethnic populations (*P*_combined_ = 2.43 × 10^−5^). Notably, expression quantitative trait loci (eQTL) data revealed that rs170183 was correlated with a decline in CLDN14 expression in both lymphoblastoid cell lines and T cells (*P*_adj_ = 0.003 and 0.016, respectively). In conclusion, our study identified a novel PBC susceptibility variant that has been shown to be strongly associated with BMD, highlighting the potential of pleiotropy to improve gene discovery.

Primary biliary cirrhosis (PBC) is the most common autoimmune liver disease that approximately affects 1 in 1000 women over the age of 40^1^,^2^. It is characterized by nonsuppurative destructive cholangitis and the production of specific anti-mitochondrial antibody (AMA)^3^. Like most other autoimmune disorders, PBC has a strong genetic component, with an estimated relative sibling risk of 10 and high familial clustering^4^. Previous studies have established that the major genetic component of PBC lies in human leukocyte antigen (HLA) region^5^,^6^,^7^. To date, Genome-wide association studies (GWAS) and fine-mapping studies have also identified multiple PBC risk loci outside HLA^8^,^9^,^10^,^11^,^12^. Despite these advances, the majority of the genetic factors underlying susceptibility to PBC remain unknown^13^.

A recent Immunochip analysis in European populations identified a novel PBC locus located upstream of the TNFSF11 gene, which encodes RANKL^14^. Interestingly, GWAS studies have shown association of this gene with bone mineral density^15^,^16^,^17^,^18^,^19^. RANKL plays a crucial role in bone resorption, osteoclast development and function^20^. Moreover, RANKL is implicated in the immune system, where it is expressed in T cells and is also involved in dendritic cell maturation^21^. In addition, *MAPT*, another gene associated with BMD also confers risk of PBC^12^,^18^. This coincides with the observation from numerous GWAS studies that many genetic variants appear to have pleotropic effects^22^.

Epidemiological and clinical studies have shown that people with PBC have an increased risk of developing osteoporosis and fracture^23^,^24^,^25^. Therefore, the epidemiological evidence together with the observed GWAS loci common to both PBC and BMD have led us to hypothesize that the comorbidity of PBC and osteoporosis may be due to shared genetic risk factors.

In the current study, we examine whether the genetic variants previously shown to be associated with BMD at genome-wide significance level also predispose to PBC. Using two European PBC GWAS data sets and a Chinese Han PBC cohort (total n = 10229), we identified a novel variant in *CLDN14* that is likely to have pleiotropic effect on PBC and BMD.

## Results

### Association analysis of BMD SNPs with PBC in two GWAS cohorts (Stage 1)

**Figure 1:**
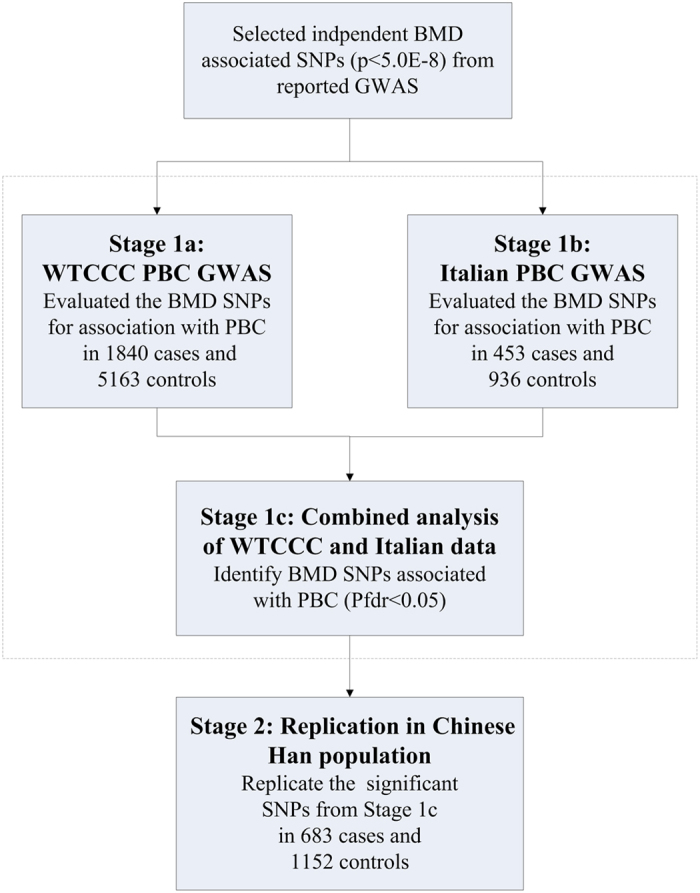
Study design of the association analysis in PBC cohorts.

We investigated 72SNPs significantly associated with BMD (p < 5 × 10^−8^) using the WTCCC PBC GWAS and Italian PBC GWAS data (Fig. 1). Association tests used an additive model and logistic regression to adjust for sex and population substructure in each cohort. The association results for each SNP were then combined by meta-analysis using inverse-variance method assuming a fixed-effect model (Supplementary Table 1). The combined analysis identified three SNPs (rs9533090, rs1864325 and rs170183) meeting a significance threshold after correction for multiple testing (Table 1). For all the three SNPs, the alleles associated with reduced BMD increased the risk of PBC. These included SNPs in two genes previously associated with PBC^12^,^14^: rs1864325 (OR = 1.27, *P*_fdr_ = 1.58 × 10^-5^) in an intron of *MAPT* gene at 17q21.1, and rs9533090 (OR = 1.20, *P*_fdr_ = 4.14 × 10^−5^) located between *AKAP11* and *TNFSF11* at 13q14. The third SNP, rs170183 (OR = 1.14, *P*_fdr_ = 0.015) located in an intron of *CLDN14* is a novel PBC association.

**Figure 2:**
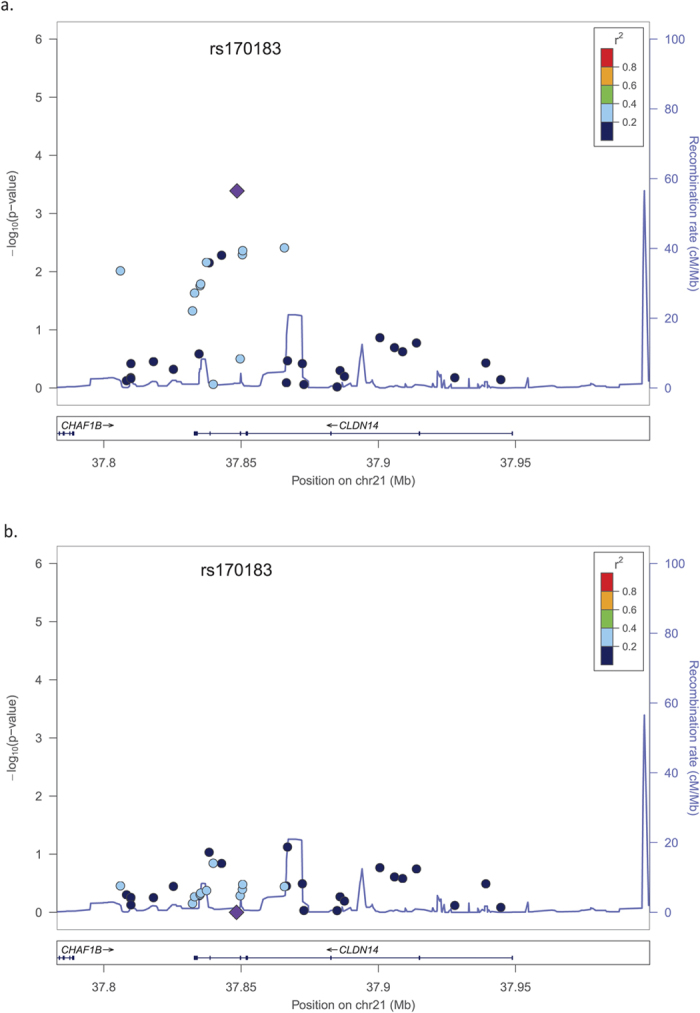
Association results from combined analysis of two European PBC GWAS data sets for the CLDN14 locus before (**a**) and after (**b**) condition on rs170183. The peak SNP, rs170183, is represented by a large purple diamond and its correlated SNPs are shown by a color gradient from red (high LD) to blue (low LD). Blue peaks represent recombination rate (1000 Genomes EUR).

In previous PBC association studies, rs3862738 and rs17564892 were the lead SNPs of *TNFSF11* and *MAPT* loci, respectively. We next examined whether the SNPs (rs9533090 and rs1864325) identified in the current study were independent signals at these two loci. Rs17564829 and rs1864325 were in complete linkage disequilibrium (r^2^ = 1). However, the SNP rs9533090 remained significant after conditional analyses adjusting for rs3862738, suggesting rs9533090 was an independent association signal. To search for potential independent signals at *CLDN14* locus, we performed conditional logistic regression analysis, including rs170183 as a covariate, using the two PBC GWAS datasets. However, this analysis did not uncover any additional independent signals (Fig. 2).

### Novel genetic variant of PBC in a Chinese population (Stage 2)

The three significantly associated SNPs from stage 1 were taken forward for replication in an independent Chinese Han PBC cohort. The novel SNP, rs170183 was the only variant that was successfully replicated in this additional cohort (OR = 1.23, *P* = 0.015, Table 2). The combined analysis, based on 10229 individuals from three independent cohorts, demonstrated a strong association of rs170183 with PBC (*P*_*combined*_ = 2.43×10^−5^). It was noteworthy that rs9533090 and rs1864325 showed substantial differences in allele frequencies between Europeans and Chinese (~50% v.s. ~8%, ~70% v.s. ~0%, respectively).

### Association of the BMD genetic risk score with PBC status in GWAS datasets

Although we did not identify many risk loci shared between PBC and BMD, there could still be variants with small effects that cumulatively account for a substantial proportion of disease susceptibility. Thus, we developed a polygenic risk score evaluating the aggregate effects of BMD associated variants in prediction of PBC status in WTCCC and Italian GWAS data. For each individual, a genetic risk score composed of 72 variants was calculated to evaluate the risk of disease. No significant association between BMD-derived genetic risk score and PBC was found in the two data sets (data not shown).

### Functional analysis of rs170183 in silico

To explore potential causal variants with deleterious functions in coding regions, we checked SNPs in strong LD with rs170183 (r^2^ > 0.9) using Haploreg v2.0. There were no variants in LD with rs170183 in the coding sequence of *CLDN14*.

**Figure 3:**
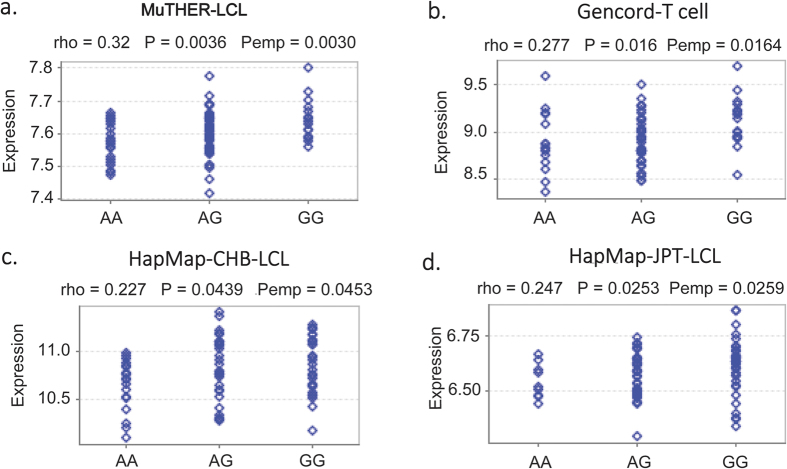
SNP rs170183 and CLDN14 expression analysis using Genevar software in (**a**) MuTHER lymphoblastoid cell line samples, (**b**) Gencord T cell samples, (**c**) HapMap3 CHB LCL samples and (**d**) HapMap3 JPT LCL samples. Pemp was calculated from 10000 permutations implemented in Genevar.

To examine how rs170183 contributes to disease susceptibility, we explored the potential biological function of rs170183. Notably, rs170183 was located in the binding motif for HNF4, a nuclear receptor protein widely expressed in liver. Furthermore, expression quantitative trait locus (eQTL) analysis using Genevar database^26^ revealed a significant effect of rs170183 on CLDN14 expression in the lymphoblastoid cell lines (LCL) based on 156 European twins from MuTHER resource^27^, with the risk allele associated with lower expression of CLDN14 (P_adj_ = 0.003, Fig. 3). Subsequently, we used data from HapMap3 and Gencord implemented in Genevar to validate the results. We found a significant correlation between rs170183 and CLDN14 gene expression levels in T cells derived from umbilical cords of 75 Gencord individuals (P_adj_ = 0.016)^28^. The analysis based on HapMap3 project also showed a moderate effect on gene expression in Asian populations (80 CHB and 82 JPT) in LCLs^29^. It is noteworthy that CLDN14 expression in peripheral blood monocytes was higher in females with high hip BMD than females with low hip BMD^15^.

## Discussion

A recent study revealed that ~5% of genome-wide significant SNPs and ~17% of genes included in NHGRI had cross-phenotype effects, suggesting the abundant pleiotropic effects on human complex traits^30^. Furthermore, several genetic pleiotropic approaches have recently shown encouraging results in capturing some of the missing heritability, especially across autoimmune diseases and neuropsychiatric disorders^31^,^32^,^33^. Here, we tested for an association between PBC and the genome-wide significant SNPs for BMD. We confirmed two known shared loci, *TNFSF11* and *MAPT*, using European GWAS data sets. Our analysis highlighted a novel variant within the *CLDN14* gene that showed consistent evidence of association with PBC in the individuals of Chinese Han as well as European ancestry.

Although we observed some shared genetic loci between PBC and BMD, we found no association between the BMD-derived GRS and PBC status, implying that BMD and PBC have distinct genetic architectures. Therefore, the observed epidemiological correlation between reduced BMD and PBC risk is likely to depend on secondary effects or shared environmental factors.

*CLDN14*, a member of the claudin family, encodes an integral membrane protein and a component of tight junction strands. Claudins act as crucial factors for epithelial barrier and transport, and therefore are considered the most important components of tight junction^34^. Disruption of tight junctions in canaliculi and bile duct participate in the pathogenesis of cholestatic liver diseases such as PBC and PSC^35^,^36^. The mutations in claudin 1 gene were reported to cause a recessive neonatal ichthyosis sclerosing cholangitis syndrome, in which a bile leakage through tight junctions of hepatocellular and biliary cells was found^37^. Claudin 2 deficiency altered paracellular permeability required for proper regulation of bile flow, which increased susceptibility to gallstone disease in mice^38^. Claudin 15-like b was found to play an important role in intrahepatic bile duct morphogenesis in zebra fish^39^. The mutations in *TJP2* gene, encoding tight junction protein2, disrupted tight junctions and led to severe cholestatic liver disease^40^. Whereas very little is known regarding the role of claudin 14 in regulation of tight junction in liver, microarray analysis found that though widely expressed, CLDN14had highest expression in liver, ( http://www.biogps.org/). In addition, the variants in *CLDN14* were suggested to be associated with kidney stone and reduced BMD^15^,^41^.

The association at the newly identified SNP rs171803 did not show heterogeneity among UK, Italian and Chinese samples (*I*^2^ = 0). However, we did observe dramatic discrepancies in the allele frequencies of rs9533090 and rs1864325 between Europeans and Chinese. Accordingly, these two variants were not replicated in the Chinese cohort. This is not surprising in that recent GWAS studies of PBC from European and Japanese populations have already indicated that PBC is characterized by genetic heterogeneity. Many PBC risk loci reported in European populations were not validated in Japanese GWAS, including *IL12A*, *SPIB* and *TNFRSF1A*. On the other hand, *POU2F1* and *TNFSF15*, identified in Japanese GWAS, were not associated with PBC in European populations^11^. Additionally, a large-scale candidate gene analysis in Chinese Han cohort showed divergence in susceptibility loci among Chinese, Japanese and Europeans^42^. Therefore, the complex mode of genetic heterogeneity in PBC underlines the need for a systematic GWAS in Chinese population to further dissect the genetic architecture of PBC.

This study had several limitations. First, we collated the SNP list of the most significant BMD loci, however, they were probably only a subset of the disease risk variants. Thus, the analysis may overlook some variations that have small or modest effects. Second, BMD levels were not available for the individuals in the PBC cohorts, so we were not able to examine the effects of the risk variant on BMD level and PBC risk in the same individual. Third, although the eQTL analysis showed that rs178103 was correlated with the expression of CLDN14, there was no direct evidence that rs178103 is a causal variant that regulates gene expression. Therefore, the future research will be warranted to define the functional implications of this variant. Fourth, the candidate SNPs tested in the Han Chinese were selected based on the European GWAS results and may not have captured risk haplotypes (if present) in disparate population groups. Finally, genetic variations in the sex chromosomes were not considered in the current study. In some recent findings, enhanced rates of X chromosome monosomy and Y chromosome loss have been observed in PBC patients^43^,^44^,^45^.

In conclusion, we identified a new locus, rs170183 in *CLDN14* at 21q22.13, associated with PBC in ethnically diverse populations. Further evaluation of the risk variant and the gene may help elucidate the mechanism of co-manifestation of PBC and osteoporosis. Our findings indicate searching for pleiotropic genes may present an important opportunity for identifying more of the missing heritability of the complex traits.

## Materials and Methods

### Study Design and Subjects

We used a two-stage design to analyze data from three independent cohorts (Fig. 1). Stage 1 consisted of combined analysis of two independent genome-wide association data sets: Welcome Trust Case Control Consortium (WTCCC) PBC GWAS (1840 PBC cases and 5163 population controls, stage 1a)^10^; Italian PBC GWAS (453 cases and 936 controls, stage 1b)^9^. The WTCCC data set was obtained from the Wellcome Trust Case Control Consortium official website ( http://www.wtccc.org.uk/). The Italian data set was obtained from the database of Genotypes and Phenotypes (dbGaP, http://www.ncbi.nlm.nih.gov/gap) through accession number phs000444.v1.p1. Details about sample characteristics, genotyping and quality control can be found in the original papers. All experiments were approved and in accordance by the Human Subjects and Ethics Committee of the State Key Laboratory for Oncogenes and Related Genes, Division of Gastroenterology and Hepatology, Renji Hospital, School of Medicine, Shanghai Jiao Tong University, Shanghai Cancer Institute, Shanghai Institute of Digestive Disease. Informed consent was obtained from all subjects.

We conducted replication analysis (stage 2) involving 685 PBC cases and 1152 controls from South China. The Chinese PBC cases were 86% female and mean age of 53.8 years (SD: 11.6). The diagnosis of PBC was based on the following three criteria: (1) abnormal liver biochemistry (one or more of bilirubin, alanine transaminase, aspartate transaminase, alkaline phosphatase or gamma-glutamyl transferase above the upper reference level); (2) a positive test for antimitochondrial antibodies (titer 1:40 or greater); or (3) a liver biopsy with histological abnormalities consistent with PBC. The patients fulfilled at least two of the three criteria were included in the study. The control data set was obtained from a previous GWAS study, in which the controls were recruited in the same region as was the PBC subjects (female: 803, male: 349; mean age: 59.1 years, SD: 10.2)^46^. Written consent forms were obtained from all the subjects. All the subjects were of Chinese Han origin by self-report.

### SNP Selection and Quality Control

We selected SNPs that showed significant association with BMD according to genome-wide association studies catalog (available at www.genome.gov/gwastudies and accessed in December 2014). Eighty-nine SNPs met genome-wide significance (*P* < 5.0 × 10^−8^) in at least one previously published Bone mineral density GWAS or meta-analysis of GWAS. Of the 89 SNPs, 25 were genotyped directly in two PBC GWAS datasets. The SNPs not genotyped were pre-phased by SHAPEIT and then imputed by IMPUTE2, using 1000 Genomes release 20101123 reference panel^47^,^48^. Imputed genotypes with posterior probability <0.9 and SNPs with info score <0.8, minor allele frequency <1%, HWE <0.00001 were removed. The data were also filtered for SNPs with <90% genotyping rate and individuals with <90% genotyping rate. One imputed SNP (hg18_chr11:27369242) with low minor allele frequency (<1%) and three imputed SNPs (rs884205, rs17040773 and rs13204965) with low genotyping rate (<90%) in Italian data set were excluded from further analysis, and no proxies were available for them. For the SNPs that were in high linkage disequilibrium (r^2^ > 0.9) with each other in each locus, only one SNP that was either directly genotyped or imputed with higher confidence was retained in the study. Thus, 13 SNPs were replaced by their proxies. After quality control, a total of 72 independent SNPs were taken forward for the association analysis and genetic risk score construction using WTCCC and Italian PBC GWAS data.

Principal components analysis was then applied to the two PBC GWAS datasets to correct for population stratification^49^. The details have been described elsewhere^50^. The first four principal components were used as covariates in the WTCCC cohort, while the first principal component was included as covariate in the Italian data set.

### SNP Genotyping

We selected the associated variants from Stage I to replicate in the Chinese cohort. The three candidate SNPs and 20 SNPs for another projects met the Sequenom design standards, and were genotyped using the Sequenom iPLEX system. Genotyping results were processed with the MassARRAY Analyzer 4. Individuals with <90% genotyping, SNPs with <90% genotyping or HWE <0.00001 were filtered.

### Statistical Analysis

#### Association analysis

Case-control association tests were conducted using logistic regression analysis implemented in PLINK v1.07 ( http://pngu.mgh.harvard.edu/ ~ purcell/plink/)^51^. The association tests in GWAS datasets were adjusted by the principal components and sex. We used the GWAMA (v2.1) to perform meta-analysis of the two GWAS datasets using inverse-variance method based on a fixed-effect model^52^. Multiple testing with the false discovery rate method was conducted in R software v2.15 ( http://www.r-project.org/).

#### eQTL analysis

We used MuTHER pilot data from lymphoblastoid cell lines (LCL) for eQTL analysis using GENEVAR (v3.3.0)^26^. Spearman’s rank correlation coefficient was performed to estimate the association between SNP genotypes and gene expression. Adjusted P values were generated from 10000 permutations implemented in GENEVAR software. To validate the results in independent datasets, we also used genotype and gene expression data from the Geneva Gencord and HapMap 3 projects.

#### Functional annotation

The regulatory elements overlapped with rs170183 were annotated with HaploReg v3 ( http://www.broadinstitute.org/mammals/haploreg/)^53^.

#### Genetic risk score construction

The genetic risk score was constructed in the two PBC GWAS datasets using the 72 SNPs associated with BMD at genome-wide level of significance (*P* < 5 × 10^−8^). We calculated the GRS by simply summing risk allele counts and dividing them by non-missing SNPs for each individual.

## Additional Information

**How to cite this article**: Tang, R. *et al.* A Common Variant in CLDN14 is Associated with Primary Biliary Cirrhosis and Bone Mineral Density. *Sci. Rep.* **6**, 19877; doi: 10.1038/srep19877 (2016).

## Supplementary Material

Supplementary Information

## Figures and Tables

**Table 1:** Top BMD SNPs associated with PBC status in combined analysis of WTCCC GWAS data and Italian GWAS data at Pfdr <0.05.

SNP	Chr.	Position (hg19)	Nearby Gene	Effect on BMD	PBC RA	WTCCC data		Italian data		Combined analysis		
						OR (95% CI)	*P*	OR (95% CI)	*P*	OR	*P*	*P*_*fdr*_
rs9533090	13	42951449	TNFSF11	T↓spine, LSBMD	T	1.21 (1.11–1.31)	9.32E-06	1.20 (1.01–1.42)	0.043	1.20 (1.12–1.30)	1.15E-06	4.14E-05
rs1864325	17	43977827	MAPT	T↓LSBMD	T	1.25 (1.13–1.38)	1.03E-05	1.34 (1.09–1.65)	0.0052	1.27 (1.16–1.39)	2.19E-07	1.58E-05
rs170183	21	37848334	CLDN14	A↓hip, FNK	A	1.12 (1.03–1.22)	0.0068	1.23 (1.03–1.46)	0.023	1.14 (1.06–1.23)	6.25E-04	0.015

The association tests were performed using logistic regression adjusted for principal components and sex.

↓, decreasing effects on BMD level.

Pfdr, false discovery rate adjusted P value based on 72 SNPs in supplementary table 1.

Chr., chromosome; RA, risk allele; OR, odds ratio.

**Table 2:** Summary statistics of the associated SNPs in the replication Chinese cohort.

SNP	Risk allele/non-risk allele	Allele frequencies (case/control)			Analysis in Chinese data	
		WTCCC	Italian	Chinese	OR (95% CI)	*P*
rs9533090	T/C	0.524/0.480	0.452/0.410	0.077/0.087	1.20 (1.01–1.42)	0.37
rs1864325	T/C	0.793/0.757	0.778/0.713	0.00073/0	1.34 (1.09–1.65)	1
rs170183	A/G	0.555/0.530	0.534/0.498	0.435/0.393	1.23 (1.03–1.46)	*0.015*

The association tests were performed using logistic regression adjusted for sex.
